# Chemical Rescue of Malaria Parasites Lacking an Apicoplast Defines Organelle Function in Blood-Stage *Plasmodium falciparum*

**DOI:** 10.1371/journal.pbio.1001138

**Published:** 2011-08-30

**Authors:** Ellen Yeh, Joseph L. DeRisi

**Affiliations:** 1 Department of Pathology, Stanford Medical School, Stanford, California, United States of America; 2 Department of Biochemistry and Biophysics, University of California, San Francisco, California, United States of America; 3 Howard Hughes Medical Institute, Chevy Chase, Maryland, United States of America; University of Georgia, United States of America

## Abstract

*Plasmodium* spp parasites harbor an unusual plastid organelle called the apicoplast. Due to its prokaryotic origin and essential function, the apicoplast is a key target for development of new anti-malarials. Over 500 proteins are predicted to localize to this organelle and several prokaryotic biochemical pathways have been annotated, yet the essential role of the apicoplast during human infection remains a mystery. Previous work showed that treatment with fosmidomycin, an inhibitor of non-mevalonate isoprenoid precursor biosynthesis in the apicoplast, inhibits the growth of blood-stage *P. falciparum*. Herein, we demonstrate that fosmidomycin inhibition can be chemically rescued by supplementation with isopentenyl pyrophosphate (IPP), the pathway product. Surprisingly, IPP supplementation also completely reverses death following treatment with antibiotics that cause loss of the apicoplast. We show that antibiotic-treated parasites rescued with IPP over multiple cycles specifically lose their apicoplast genome and fail to process or localize organelle proteins, rendering them functionally apicoplast-minus. Despite the loss of this essential organelle, these apicoplast-minus auxotrophs can be grown indefinitely in asexual blood stage culture but are entirely dependent on exogenous IPP for survival. These findings indicate that isoprenoid precursor biosynthesis is the only essential function of the apicoplast during blood-stage growth. Moreover, apicoplast-minus *P. falciparum* strains will be a powerful tool for further investigation of apicoplast biology as well as drug and vaccine development.

## Abstract 2

The only essential function of a unique plastid organelle, the apicoplast, in blood-stage *P. falciparum* is the production of isoprenoid precursors.

## Author Summary

Malaria caused by *Plasmodium* spp parasites is a profound human health problem that has shaped our evolutionary past and continues to influence modern day with a disease burden that disproportionately affects the world's poorest and youngest. New anti-malarials are desperately needed in the face of existing or emerging drug resistance to available therapies, while an effective vaccine remains elusive. A plastid organelle, the apicoplast, has been hailed as *Plasmodium*'s “Achilles' heel” because it contains bacteria-derived pathways that have no counterpart in the human host and therefore may be ideal drug targets. However, more than a decade after its discovery, the essential functions of the apicoplast remain a mystery, and without a specific pathway or function to target, development of drugs against the apicoplast has been stymied. In this study, we use a simple chemical method to generate parasites that have lost their apicoplast, normally a deadly event, but which survive—“rescued” by the addition of an essential metabolite to the culture. This chemical rescue demonstrates that the apicoplast serves only a single essential function, namely isoprenoid precursor biosynthesis during blood-stage growth, validating this metabolic function as a viable drug target. Moreover, the apicoplast-minus *Plasmodium* strains generated in this study will be a powerful tool for identifying apicoplast-targeted drugs and as a potential vaccine strain with significant advantages over current vaccine technologies.

## Introduction

The discovery of a plastid organelle, the apicoplast, in *Plasmodium* spp. (responsible for 250 million cases of human malaria each year) and other Apicomplexa parasites instantly made it a key target in the development of new therapies against these pathogens [1]–[3]. The need for new anti-malarials is particularly urgent given the documentation of developing resistance to the current last-line therapy in the deadliest species, *P. falciparum* [4]. Several features of this organelle make it both biologically fascinating and an attractive therapeutic target. The apicoplast is derived from secondary endosymbiosis of a plastid-bearing red algae and is therefore prokaryotic in origin, containing pathways that have no counterpart in the human host [3],[5],[6]. During the course of evolution, the apicoplast has lost its photosynthetic function and transferred most of its genome to the nucleus, requiring a dedicated protein targeting pathway to localize the majority of its over 500 gene products into the organelle [7],[8]. The remaining 35 kb apicoplast genome encodes ∼50 mostly housekeeping genes, including ribosome subunits, tRNAs, RNA polymerase, and a chaperone thought to be involved in protein import [1]. Despite its minimalization, the apicoplast continues to serve essential though poorly defined metabolic function(s). In *Plasmodium*, apicoplast function is necessary for both intraerythrocytic and intrahepatic development in the human host [9],[10]. Whether the apicoplast is required for sexual stage development in the mosquito is currently unknown [11]–[14].

The essential role of the apicoplast has been clearly demonstrated by the effect of antibiotics on blood-stage *P. falciparum*. Dahl et al. showed that antibiotics that inhibit prokaryotic transcription or translation, such as doxycycline, specifically blocks expression of the apicoplast genome [9],[15]. Parasites treated during first 48 h life cycle show no obvious defect from the loss of apicoplast-encoded gene products: Organelle morphology, genome replication, protein targeting, and segregation during cell division remain intact. Likewise, parasites progress normally through the ring, trophozoite, and schizont developmental stages, giving rise to daughter merozoites that successfully reinvade to establish infection of a new host cell. Instead, a curious “delayed death” phenotype is observed, whereby the deleterious effects of antibiotic inhibition accumulate in the *progeny* of treated parasites. In this 2^nd^ life cycle following antibiotic treatment, the apicoplast genome fails to replicate. Protein import function is lost. Finally, the organelle itself fails to replicate and segregate during cell division. Because the apicoplast cannot be generated de novo and must be inherited at each cell division, the failure of organelle replication and segregation in these parasites results in loss of the apicoplast in daughter cells and parasite death. Overall, antibiotic-induced delayed death begins with specific inhibition of apicoplast transcription and translation in one life cycle and ends with irreversible apicoplast loss and death in the subsequent cycle. A similar delayed death has been observed in *Toxoplasma gondii* following antibiotic treatment or transgene expression that cause apicoplast loss [16],[17].

Despite its promise as *Plasmodium*'s “Achilles heel,” the function of the apicoplast has eluded researchers in the nearly 20 years since its discovery. Without knowledge of specific proteins or pathways suitable as drug targets, particularly during the clinically symptomatic blood stage, efforts to develop apicoplast-directed therapies (beyond known antibiotics) have been stymied. An astounding 5%–10% of the nuclear genome is predicted to contain an apicoplast targeting signal, yet 70% of these apicoplast gene products are of unknown function [18]–[20]. Pathways that have been identified include those for the biosynthesis of isoprenoid precursors, fatty acids, heme, Fe-S clusters, and lipoic acid [21]. While in silico analysis has been revealing, many pathways will go undetected and the essentiality of predicted pathways throughout the parasite's complex life cycle needs to be experimentally validated. For example, inhibition by the antibiotic triclosan initially suggested that apicoplast-located type II fatty acid biosynthesis was essential in blood-stage parasites, prompting the development of fatty acid inhibitors as anti-malarials [22]. Later, genetic deletion of fatty acid biosynthetic genes definitively proved that this pathway is not required for blood stage growth and instead is critical for liver stage development [23],[24]. Unfortunately, discovery and validation of apicoplast pathways has been hampered by the limited ability to generate knockouts of essential genes, isolate the organelle, or purify Plasmodial proteins for in vitro characterization [25]–[28].

Amongst the annotated apicoplast pathways, *Plasmodium* relies on the prokaryotic MEP/DOXP/non-mevalonate pathway for synthesizing isoprenoid precursors rather than the canonical mevalonate pathway used by most other eukaryotes and all mammals (including the human host) [29],[30]. Both pathways produce isopentenyl pyrophosphate (IPP) and dimethylallyl pyrophosphate (DMAPP) as the final products, but the enzymes and chemical intermediates leading to synthesis of these compounds are entirely different. Fosmidomycin, an inhibitor of the MEP pathway, kills blood-stage parasites and has been tested in clinical trials as an antimalarial [31],[32]. Inhibition by fosmidomycin suggests that isoprenoid precursor biosynthesis is essential in blood-stage infection, although the possibility of off-pathway targets as the cause of the drug effect (as was found to be the case for triclosan) has not been ruled out [29],[33]. Furthermore, IPP and DMAPP are not an end onto themselves but rather building blocks used to synthesize small molecule isoprenoids with a host of functions or C_15_/C_20_ prenyl chains for the post-translational modification of proteins [34],[35]. Once IPP and DMAPP are exported into the parasite cytoplasm, the downstream isoprenoid products in *Plasmodium* and their function during infection are unknown.

The significance of isoprenoid precursor biosynthesis as a drug target and gateway for identifying isoprenoid products with essential functions in pathogenesis depends on a clear demonstration of its role in parasite survival. In addition to its essentiality, this pathway may represent the only direct output from the apicoplast into the cytoplasm during blood stage growth since the remaining annotated pathways function primarily for organelle maintenance, support the mitochondria, or are not essential in this stage. Given the difficulty of studying the apicoplast by traditional methods, we employed an alternative strategy using drug inhibition/chemical rescue, equivalent to genetic deletion/complementation, to establish pathway essentiality and sufficiency. Using this simple chemical genetic approach, we show that isoprenoid precursor biosynthesis is not only essential but in fact the sole essential function of the apicoplast during blood-stage growth.

## Results

### Isoprenoid Precursors Chemically Rescue Fosmidomycin Inhibition

**Figure 1:**
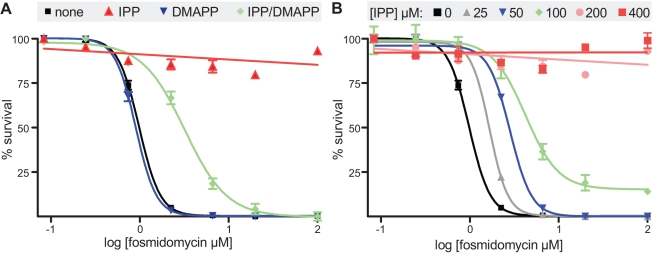
Chemical rescue of fosmidomycin inhibition with IPP precursors. (A) Growth inhibition by fosmidomycin (0–100 µM) in media supplemented with 200 µM IPP, DMAPP, or both IPP and DMAPP. (B) Concentration dependence of IPP rescue.

To investigate the specificity of fosmidomycin for the isoprenoid precursor biosynthetic pathway, we observed the effect of supplementation with isoprenoid precursors, IPP and DMAPP, on drug inhibition of blood-stage parasites. Growth inhibition of blood-stage *P. falciparum* W2 by fosmidomycin occurred with an EC_50_ = 0.98 µM (95% confidence interval = 0.93–1.03 µM; Figure 1A). When drug susceptibility was performed in media supplemented with 200 µM IPP, DMAPP, or both IPP and DMAPP, only IPP (without DMAPP) was sufficient to completely reverse the growth inhibition in the presence of up to 100 µM fosmidomycin (Figure 1A). Survival of parasites was dependent on the concentration of IPP in the media with rescue apparent at 200 µM IPP (Figure 1B). DMAPP alone or in combination with IPP had no effect or was even slightly toxic (Figure 1A). Addition of up to 2 mM 3-methyl-3-butenol, the alcohol analog of IPP lacking the pyrophosphate moiety, alone or in combination with 3-methyl-2-butenol, the alcohol analog of DMAPP, also did not rescue fosmidomycin inhibition (Figure S1). Finally, reversal of drug inhibition by addition of IPP was only seen with fosmidomycin and did not occur with chloroquine, a drug that does not target isoprenoid precursor biosynthesis (Figure S2). These findings establish that (1) fosmidomycin inhibition is specific for the isoprenoid precursor biosynthetic pathway, (2) isoprenoid precursor biosynthesis is essential for blood-stage *P. falciparum*, and (3) exogenous IPP fulfills endogenous biosynthetic function.

### IPP Supplementation Rescues Antibiotic-Induced Delayed Death

To determine whether rescue of the isoprenoid precursor biosynthesis pathway can reverse delayed death due to antibiotics, *P. falciparum* W2 parasites were treated with chloramphenicol, clindamycin, or doxycycline in the presence of IPP. As described above, treatment of blood-stage parasites with these prokaryotic transcription and translation inhibitors specifically blocks apicoplast gene expression in the first 48 h cycle leading to apicoplast loss and parasite death in the second cycle [9],[15]. As such, antibiotic treatment yielded a 48 h EC_50_ in growth assays due to nonspecific inhibition and a lower 96 h EC_50_ due to apicoplast-specific inhibition (Table 1). This shift in the EC_50_ values from 48 to 96 h is characteristic of the delayed death phenotype. By supplementing with 200 µM IPP, reversal of apicoplast-specific inhibition at 96 h by all drugs was observed with EC_50_ values reflective of only nonspecific drug effects. In fact, when addition of IPP was compared from 48–96 h or 0–96 h, IPP was only required during the second cycle consistent with a deficiency in the *progeny* of treated parasites (Table 1). Similar results were demonstrated with *P. falciparum* D10 strain, suggesting that the rescue of antibiotic inhibition by IPP is not strain-specific (Table 1).

**Table 1:** Rescue of apicoplast-specific growth inhibition by antibiotics.

Strain	Drug	EC_50_ (µM)^a^				
		48 h		96 h		
		No Rescue	+IPP	No Rescue	+IPP 0–96 h	+IPP 48–96 h
**W2**	**Chloramphenicol**	>300^b^	>300^b^	13.7 (9.7–19.4)	>300^b^	>300^b^
**W2**	**Clindamycin**	>10^b^	>10^b^	0.004 (0.002–0.006)	>10^b^	ND
**W2**	**Doxycycline**	4.9 (2.9–8.5)	5.1 (2.3–11.0)	0.3 (0.2–0.4)	3.2 (2.6–3.9)	ND
**D10**	**Chloramphenicol**	>300^b^	>300^b^	19.8 (13.6–28.8)	>300^b^	>300^b^

ND, not determined.

a Represented as mean (95% confidence interval).

b 50% inhibition above highest assayed concentration.

**Figure 2:**
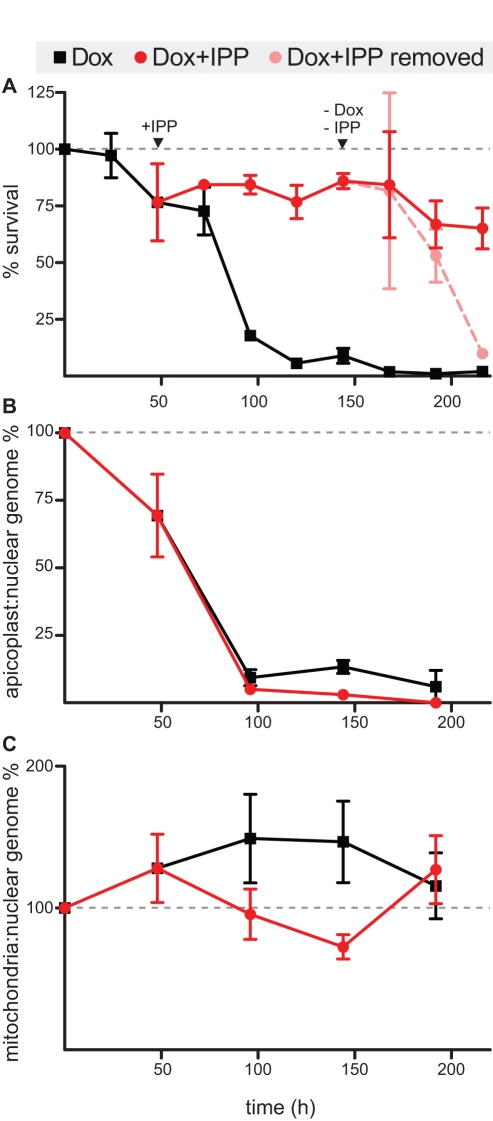
Rescue of antibiotic delayed death and apicoplast genome loss. (A) Survival of parasites over 4 life cycles treated with (1) doxycyline only, (2) doxycycline+IPP, or (3) doxycycline+IPP for three cycles followed by removal of both. Parasitemia is normalized to that of an untreated control. (B) Apicoplast∶nuclear and (C) mitochondria∶nuclear genome ratio of doxycycline only and doxycycline+IPP treated parasites over the same time course. Genome ratios are normalized to an untreated control. Data from three separate passages are shown.

Blood-stage parasites were carried through several life cycles with simultaneous antibiotic treatment and IPP rescue to determine (1) any significant growth defects and (2) the dependence on further supplementation with IPP (after removal of the antibiotic) of the surviving parasites. As shown in Figure 2A, the doxycycline-treated, IPP-rescued strain showed parasitemia ≥65% of that seen in the untreated strain throughout the treatment/rescue course. (Given the narrow concentration range between apicoplast-specific and nonspecific inhibition for doxycycline, some decreased growth due to non-specific inhibitory effects is expected at the drug concentration used.) Moreover, the rescued strain was carried for a total of 26 days with IPP supplementation but in the absence of antibiotics with no diminishment in growth capacity. In contrast, doxycycline-treated parasites without added IPP quickly died after the 2^nd^ cycle of treatment with undetectable parasitemia by the end of the 3^rd^ cycle (Figure 2A). Significantly, removal of IPP and doxycycline from the media at the start of the 4^th^ cycle results in a rapid decline in parasitemia of the rescued parasites (Figure 2A). With further passage in media lacking both IPP and doxycycline, the parasitemia of rescued parasites became undetectable. Again similar results could be demonstrated with *P. falciparum* D10 strain or treatment with chloramphenicol (Figures S3 and S4). These results show that antibiotic-treated parasites rescued by IPP supplementation have no gross growth defect but are entirely dependent on exogenous IPP for continued growth. We cannot rule out more subtle growth defects that would be difficult to assess by comparison of parallel cultures.

### Antibiotic-Treated, IPP-Rescued Parasites Lose Their Apicoplast Genome

IPP rescue of death following antibiotic treatment could be due to either (1) blocking the deleterious effects of antibiotic treatment to cause apicoplast loss or (2) compensating for the loss of the apicoplast. The irreversible dependence of the rescued parasites on IPP (even after removal of the antibiotic) suggests the latter—that apicoplast loss occurs but is chemically complemented by exogenous IPP. We sought to confirm whether the sequelae of apicoplast dysfunction that occurs following treatment with antibiotics (reviewed above) also bears out in IPP-rescued parasites [9]. A hallmark of organelle dysfunction is the loss of the apicoplast genome [9],[16]. We used quantitative PCR for target genes on the apicoplast, mitochondria, and nuclear genomes to monitor the ratio of organelle: nuclear genomes during the course of antibiotic treatment and chemical rescue. Figure 2B demonstrates a marked decline in the apicoplast∶nuclear genome ratio after the 2^nd^ cycle in all antibiotic-treated parasites regardless of supplementation with IPP. At the end of the 4^th^ cycle, the ratio is reduced by at least 100-fold. In contrast, no such decline is noted in the mitochondria∶nuclear genome ratio (Figure 2C). Rescued strains carried out for a total of 26 days continued to show undetectable apicoplast genome and detectable mitochondria and nuclear genomes. Thus, IPP-rescued parasites undergo a specific and irreversible loss of the apicoplast genome without concomitant loss of the nuclear or mitochondrial genomes, yet these parasites continue to be viable.

### Antibiotic-Treated, IPP-Rescued Parasites Lose Protein Import Function

**Figure 3:**
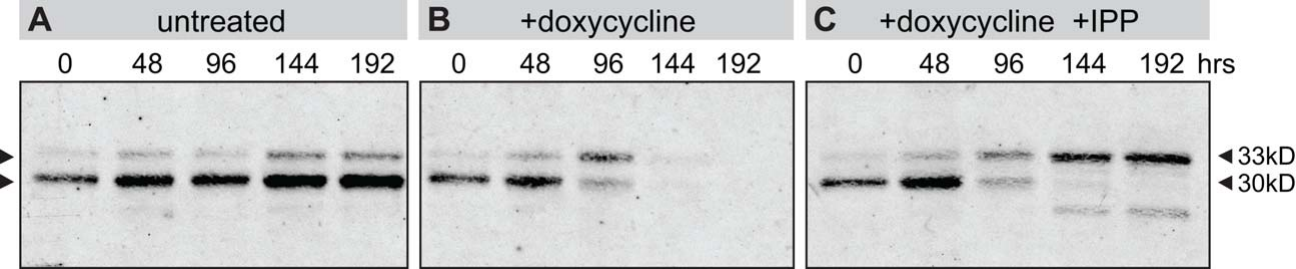
Loss of protein processing of apicoplast-targeted proteins in antibiotic-treated, rescued parasites. Immunoblot using anti-GFP shows a time course of apicoplast-dependent protein processing of apicoplast-targeted GFP in (A) untreated, (B) doxycycline-treated, and (C) doxycycline+IPP treated parasites.

A critical function of the apicoplast, required for the maintenance of its proteome, is the import of nuclear-encoded proteins into the organelle. A bipartite *N*-terminal sequence consisting of a signal sequence and a transit peptide is required to target proteins to the organelle [8]. Upon import into the apicoplast, the transit peptide is cleaved to produce a mature protein [8]. Protein processing is therefore a marker of successful protein import into the apicoplast. We used a transgenic D10 strain expressing GFP fused to an *N*-terminal apicoplast targeting sequence (ACP_L_-GFP) to assess apicoplast protein processing during the course of antibiotic treatment and IPP rescue [8]. The 33 kDa full-length GFP was cleaved to produce a predominant 30 kDa mature protein in untreated parasites (Figure 3A). Parasites treated with doxycycline only began to lose protein processing function during the 2^nd^ cycle as seen in the increased accumulation of full-length protein at 96 h but do not survive beyond this cycle (Figure 3B). When doxycycline treatment was rescued with IPP, surviving parasites showed successive loss of protein processing with each treatment cycle such that only preprocessed GFP was detectable at 144 h, the start of the 4^th^ cycle (Figure 3C). A smaller, previously described degradation band also became apparent in the rescued parasites [8]. The absence of protein processing activity indicates a loss of the critical protein import function of the apicoplast in these rescued parasites.

### Antibiotic-Treated, IPP-Rescued Parasites Lack an Apicoplast

**Figure 4:**
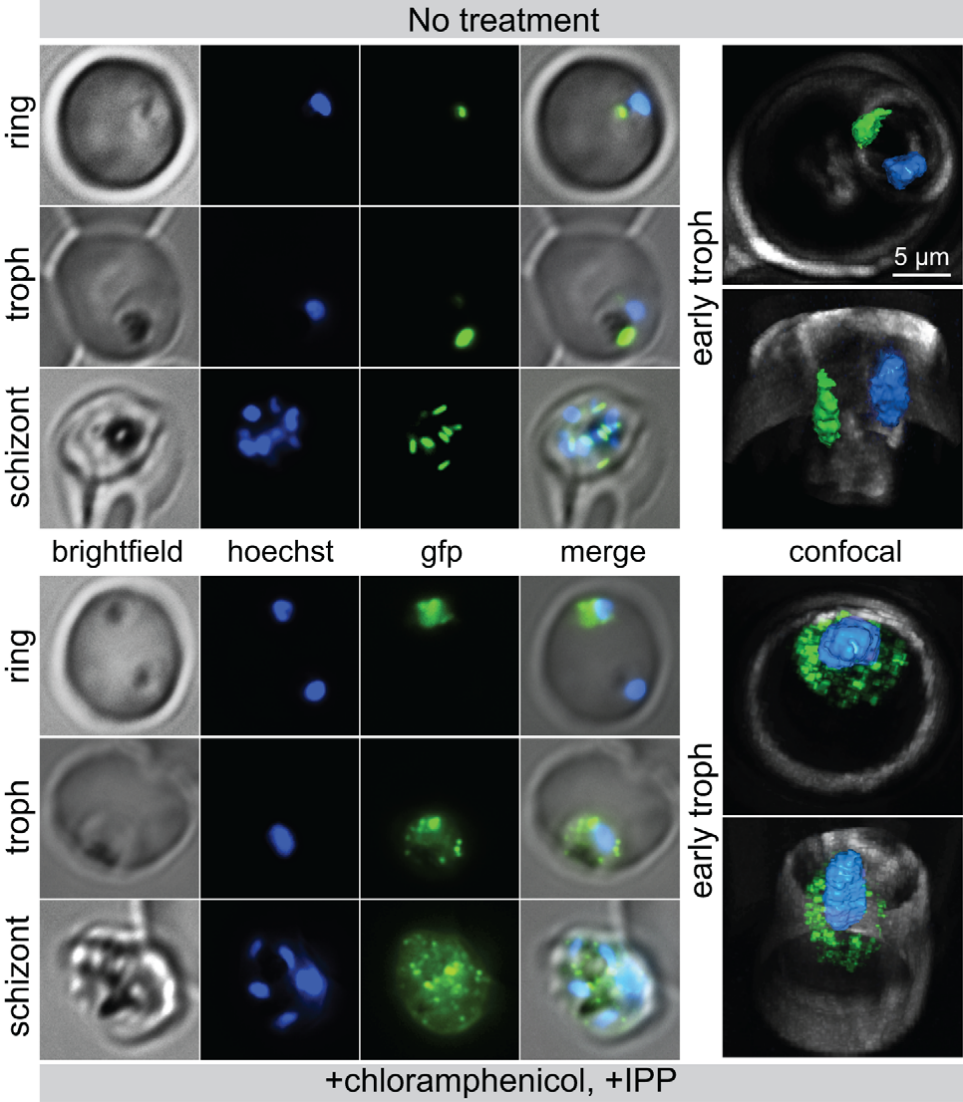
Loss of localization of apicoplast-targeted proteins in antibiotic-treated, rescued parasites. Widefield epifluorescence microscopy for apicoplast (ACP_L_-GFP) and nucleus (Hoescht 33342) comparing ring, trophozoite, and schizont in untreated parasites and chloramphenicol+IPP treated parasites. To right, confocal 3-D reconstruction of untreated parasites and chloramphenicol+IPP treated early trophozoite stage parasites.

The final outcome of antibiotic treatment is a failure of apicoplast replication and segregation during cell division, resulting in loss of the organelle and death [9]. The loss of the genome and protein import function strongly suggests that parasites that survive antibiotic treatment are in fact apicoplast-minus. Localization of GFP in the D10 ACP_L_-GFP strain was used to visualize the apicoplast. As expected, GFP localizes to a discrete structure in the parasite in untreated cells (Figure 4 and Video S1). In contrast, in parasites that have been rescued from antibiotic death, GFP loses this discrete apicoplast localization and becomes diffuse (Figure 4). Confocal images show that numerous foci of GFP are scattered throughout the cytoplasm (Figure 4 and Video S2). The largest foci measure >200 nm, so these collections of GFP are less likely to be cytoplasmic protein aggregates but instead may represent vesicles containing protein. Combined with the absence of the apicoplast genome and protein import function, the loss of GFP localization indicates the absence of the apicoplast itself to which it is normally targeted.

## Discussion

**Figure 5:**
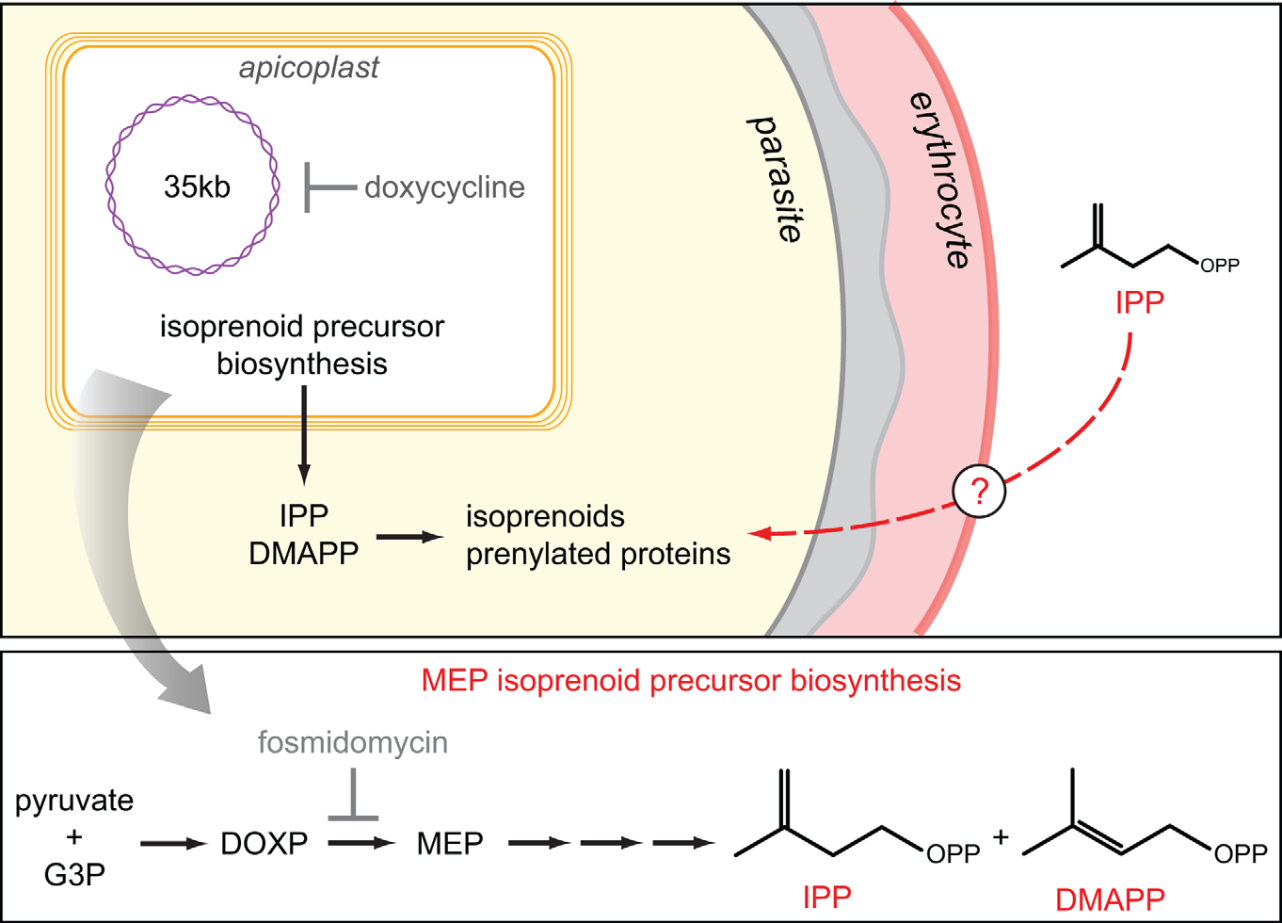
Model of apicoplast function. (Top) The essential function of the apicoplast is the production of isoprenoid precursors, IPP and DMAPP, which are exported into the cytoplasm and used to synthesize small molecule isoprenoids and prenylated proteins. Parasites that are unable to synthesize isoprenoid precursors either due to inhibition of the biosynthetic pathway by fosmidomycin or loss of the apicoplast following doxycycline inhibition can be chemically rescued by addition of exogenous IPP (red). The exogenous IPP enters the host cell through unknown membrane transporters and fulfills the missing biosynthetic function. (Bottom) Reaction scheme for MEP pathway biosynthesis of IPP and DMAPP with the enzymatic step inhibited by fosmidomycin indicated.

Until now, the mystery of apicoplast function has been a critical gap in our understanding of malaria pathogenesis. Our findings demonstrate that the production of isoprenoid precursors is the only essential function of the apicoplast during asexual blood-stage *P. falciparum* growth (Figure 5). This surprising revelation has several important implications and invites a host of new questions. Because isoprenoid precursors are building blocks to synthesize cellular isoprenoid products with diverse functions, their key role now gives added urgency to the elucidation of these products and their downstream functions. At least one essential prenylated product is ubiquinone, a component of the mitochondrial electron transport chain. There are certainly other essential, as-yet-unidentified isoprenoid products since transgenic parasites which express yeast dihydroorotic acid dehydrogenase and no longer require their electron transport chain are still susceptible to fosmidomycin and antibiotics and could be rescued with IPP supplementation (unpublished data) [36]. Possible isoprenoid products include dolichols involved in protein *N*-glycosylation which have been detected in *Plasmodium* and prenylated proteins, such as Rab homologs required for vesicular trafficking and a recently identified tyrosine phosphatase [37]–[41].

The current findings also imply that several annotated apicoplast pathways are in fact non-essential. Amongst both identified pathways and the 70% of apicoplast gene products with unknown function, only isoprenoid precursor biosynthesis and any pathways supporting this function in blood-stage parasites (including those required for organelle maintenance and replication) are essential and therefore viable apicoplast drug targets [20]. Assertions that type II fatty acid and, by implication, acetyl-CoA biosynthesis were essential apicoplast functions during blood-stage growth have already been disproven [23],[24],[42]. A parasite-derived pathway for heme biosynthesis contains steps that occur in the apicoplast, mitochondria, and cytosol. Our results strongly imply that blood-stage parasites do not depend on de novo heme biosynthesis using this pathway but instead rely on an extrinsic de novo pathway utilizing imported host enzymes or salvage of heme from the host by an unidentified mechanism [43],[44]. Still other pathways such as Fe-S cluster biosynthesis supply cofactors for enzymes within the organelle but are not exported outside the organelle. These pathways become “non-essential” when the need for organelle maintenance is removed.

The complexity of the organelle and the simplicity of its blood-stage function pose an obvious contradiction. Approximately 5%–10% of the *Plasmodium* genome is predicted to encode apicoplast-targeted gene products (although the localization and/or function of the majority of these gene products have not been validated) [20]. In order to import these proteins into the apicoplast, parasites utilize a dedicated protein trafficking pathway [7],[8]. In addition, the organelle undergoes complex morphological development during blood stage growth, requiring cellular machinery to faithfully replicate and segregate the organelle at every cell division [10]. Why are such huge resources consumed to maintain a single essential function? First, while the function of the apicoplast is limited during the blood stage, the need for more extensive organelle function during other developmental stages may dictate its maintenance in intraerythrocytic parasites as the organelle cannot be generated de novo. Fatty acid biosynthesis, for example, is an essential apicoplast function in liver stage parasites [23],[24]. Second, *Plasmodium* may have been evolutionarily trapped in its bondage to the apicoplast. Having acquired the plastid early in its evolution, it may have been unable to dispense of it even after adopting an increasingly parasitic lifestyle due to the transfer of even a few essential functions to the organelle. In any case, this imbalance emphasizes the value of targeting housekeeping pathways involved in organelle maintenance and replication to interfere with its function.

An important consideration is whether our findings accurately reflect in vivo growth requirements of parasites during infection. Specifically, are there essential metabolites supplemented in culture which could not be acquired during in vivo growth and instead must be biosynthesized by the apicoplast? While parasitized RBCs during infection use human plasma as a source of extracellular nutrients, our cultures were grown in RPMI medium 1640 supplemented with purified serum substitute, Albumax. We found that Albumax could be replaced with 10% human serum with no effect on the survival of apicoplast-minus parasites in the presence of IPP (Figure S5). RPMI medium contains salts, 20 amino acids, 11 vitamins, 4 other organic molecules, and glucose. The acquisition and biosynthesis of these nutrients by blood-stage *Plasmodium* and their essentiality for intraerythrocytic growth based on available evidence is shown in Table S1. In general, blood-stage *Plasmodium* biosynthesizes very limited amounts of just 3 amino acids and is dependent on amino acids from either (1) hemoglobin degradation or (2) acquisition from patient plasma through newly established permeation pathways in the infected red cell [45]–[47]. Similarly, current knowledge of *Plasmodium* metabolism also suggests that the remaining organic metabolites found in RPMI medium are biosynthesized by non-apicoplast pathways or can be acquired from the host red cell or patient plasma [46],[48]–[50]. Consequently, we believe that our findings can be extrapolated to in vivo requirements for the apicoplast to support parasite growth and development. At the very least, our results define a very minimal set of potential metabolites (IPP and components found in RPMI 1640 medium) that could be biosynthesized in the apicoplast. We cannot, however, rule out additional apicoplast functions (other than those required for growth) that would not be revealed in our blood culture system, such as functions required for immune evasion.

Several aspects of the chemical rescue with isoprenoid precursors are notable. During chemical rescue, exogenous IPP could enter the parasite through permeation pathways established in the parasitized erythrocyte or other uncharacterized membrane transporters [46],[51]. The RBC is largely metabolically inactive and unlikely to have significant ongoing isoprenoid precursor biosynthesis via the host mevalonate pathway or stores of these metabolites [52]. It is also unlikely that these high-energy pyrophosphorylated molecules would accumulate to appreciable levels in plasma (200 µM was required for rescue in our experiments). Consistent with this notion, IPP was not present in the Serum Metabolome Database (SMDB), which contains 4,229 detectable metabolites [53]. Therefore, acquisition of isoprenoid precursors in vivo by salvage of IPP from infected blood is improbable. Once in the parasite, exogenous IPP may fulfill its function in the cytoplasm with or without uptake into the apicoplast [54].

Although both IPP and DMAPP are required to synthesize isoprenoid products, supplementation with IPP alone is sufficient to fulfill endogenonous isoprenoid precursor biosynthesis, implying the presence of an IPP isomerase in the cytoplasm that converts IPP to DMAPP. This activity may be important in establishing the optimal cellular ratio of IPP to DMAPP, as toxicity was noted with increasing DMAPP concentrations in our experiments. A putative IPP isomerase has been identified in the *Plasmodium* genome [55]. A recent report suggested that geranylgeraniol, the alcohol analog of a C_20_ prenyl chain, could rescue fosmidomycin inhibition [56]. We were unable to rescue fosmidomycin inhibition with alcohol analogs of IPP and DMAPP, indicating either poor cellular penetration of the alcohols or the absence of a kinase to convert the alcohol analogs to the pyrophosphorylated and active metabolites (Figure S1). Even with conversion of geranylgeraniol to geranylgeranyl pyrophosphate in the cell, it would seem that a C_5_ building block, such as IPP, would almost certainly be required to extend the supplemented C_20_ unit for construction of polyprenyl chains, such as that found in ubiquinone, and to construct smaller prenyl chains, such as for protein farnesylation. The reported rescue with geranylgeraniol was performed at 1.5 µM fosmidomycin, which is above the concentration required for 50% growth inhibition but may be below that required for adequate inhibition of the biosynthetic pathway (since phenotypic growth inhibition can be apparent even at low levels of inhibition of the biosynthetic pathway) [56]. Therefore, the reported results may be complicated by ongoing biosynthesis of IPP and DMAPP contributing to the precursor pool. Consistent with this, neither farnesol nor geranylgeraniol was able to rescue fosmidomycin concentrations >10 µM, and both showed dose-related parasite toxicity (Figure S6). In contrast, we were able to demonstrate IPP rescue at fosmidomycin concentrations exceeding 100 µM, well above its EC_90_ for growth inhibition.

The consequences of apicoplast loss following antibiotic treatment and IPP rescue are no less intriguing. In the parasites that survive antibiotic treatment by chemical rescue, the organelle is irreversibly lost when it fails to segregate to daughter cells [9]. In these apicoplast-minus parasites, apicoplast gene products encoded in the nucleus may continue to be transcribed and translated. These products may properly shuttle into the secretory pathway but cannot be diverted to the organelle [8]. Based on the microscopy results, we hypothesize that proteins may be packaged into transport vesicles bound for the organelle but are unable to localize to the missing structure and therefore accumulate in the cytoplasm appearing as numerous foci. While we cannot rule out the presence of structural remnants of the apicoplast, the observed foci are unlikely to support apicoplast functions. Apicoplast-targeted proteins may require both cleavage of the long basic transit peptide and chaperones in the lumen of the apicoplast for proper folding. We observed that cleavage of the transit peptide from targeted proteins, a critical apicoplast function, does not occur in rescued parasites (Figure 3C).

The close physical and functional relationship between the apicoplast and the mitochondria raises the possibility that loss of the apicoplast might affect the ability of the mitochondria to replicate and divide. We were able to detect the mitochondrial genome by qPCR for the cytB3 gene and observe labeling of the mitochondria with Mitotracker by fluorescence microscopy in apicoplast-minus parasites (Figure 2C; unpublished data). Despite the loss of the apicoplast, these parasites do appear to contain mitochondria.

While the survival of apicoplast-minus *P. falciparum* invokes a slew of intriguing questions, these same parasites will be a powerful and indispensable tool for further dissection of apicoplast biology. Apicoplast-minus *P. falciparum* strains generated in this study can be used to assess organelle requirement during gametocytogenesis and mosquito stage development. These strains also provide novel avenues to identify isoprenoid products, generate conditional mutants of essential genes involved in apicoplast maintenance and replication, conduct metabolomic or proteomic profiling, and study protein trafficking to the organelle.

With regard to drug development, our chemical rescue strategy also addresses the critical deficiency of current cell growth screening assays, namely lack of knowledge of the drug target. Candidate drug hits detected in phenotypic assays can be screened for chemical rescue of the growth inhibition. The reversal of growth inhibition by IPP supplementation specifically identifies inhibitors that target pathways involved in MEP pathway function, replication, or maintenance of the apicoplast, providing a pathway-specific drug screen to aid in discovery of new classes of anti-malarials. The ability to chemically complement the cell death phenotype will prevent false leads from off-target effects, like that seen with triclosan and its misconstrued effect on type II fatty acid biosynthesis [22].

Finally, the apicoplast-minus strains dependent on IPP for continued growth are a unique and ideal candidate for an attenuated blood-stage vaccine [57],[58]. Unlike irradiated or drug-treated whole parasite vaccines, apicoplast-minus parasites would continue to develop in blood at most one cycle, including a single erythrocyte rupture and reinvasion, thereby stimulating a stronger immune response. However, judging by the effects of IPP withdrawal in culture, they would be unable to develop further in the absence of exogenous IPP (Figure 2A). Lending support to this notion, a similar “limited survival” strategy targeting the apicoplast in liver-stage parasites has proven effective as a liver-stage vaccine candidate [59]. A significant advantage of our approach is that attenuation is achieved chemically and does not require difficult or costly genetic manipulation (as is the case with genetically modified vaccine strains), thereby allowing for the possibility of incorporating circulating field strains of *Plasmodium* in a vaccine formulation [60]. There would also be very little risk of reversion as it would be extremely difficult to reacquire apicoplast function by mutation.

In summary, we believe that the current study ushers in a new era in the investigation of the apicoplast in *Plasmodium* with exciting opportunities to counteract the malarial scourge on human lives.

## Materials and Methods

### *P. falciparum* Cultures

*Plasmodium falciparum* W2 (MRA-157), D10 (MRA-201), and D10 ACP_L_-GFP (MRA-568) were obtained from MR4. Parasites were grown in human erythrocytes (2% hematocrit) in RPMI 1640 media supplemented with 0.25% Albumax II (GIBCO Life Technologies), 2 g/L sodium bicarbonate, 0.1 mM hypoxanthine, 25 mM HEPES (pH 7.4), and 50 µg/L gentamycin, at 37°C, 5% O_2_, and 6% CO_2_. For D10 ACP_L_-GFP, the media was also supplemented with 100 nM pyrimethamine (Sigma).

For passage of antibiotic-treated, IPP-rescued parasites, the media was supplemented with 1.5–2 µM doxycycline or 50 µM chloramphenicol. 48 h after initiation of antibiotic treatment, rescued strains were supplemented with 200 µM IPP (Isoprenoids LC) for continuous passage. For comparison of growth between different treatment conditions, cultures were carried simultaneously and handled identically with respect to media changes and addition of blood cells.

### Drug Susceptibility Assays

Growth assays were performed in 96-well plates containing serial dilution of drugs in duplicate or triplicate. Media was supplemented with IPP or DMAPP as indicated. To determine the EC_50_ of fosmidomycin (Invitrogen), growth was initiated with ring-stage parasites (synchronized with 2.5% sorbitol treatment 48 h prior) at 1% parasitemia (0.5%–2% hematocrit). Plates were incubated for 72 h. To determine the EC_50_ of antibiotics at 48 h, growth was initiated at 1% parasitemia and incubated for 48 h. To determine the EC_50_ of antibiotics at 96 h and observe the delayed death phenotype, cultures were initiated at 0.2% parasitemia, 75% of the media was exchanged at 48 h, and plates were incubated for an additional 48 h (total 96 h). For all assays, growth was terminated by fixation with 1% formaldehyde and parasitized cells were stained with 50 nM YOYO-1 (Invitrogen). Parasitemia was determined by flow cytometry. Data were analyzed by FlowJo, and EC_50_ curves plotted by GraphPad Prism.

### Quantitative Real-Time PCR

Parasites from 200 µL of culture were isolated by saponin lysis followed by PBS wash to remove extracellular DNA. DNA was purified using QiaAMP blood kits (Qiagen). Primers were designed to target genes found on each organelle or nuclear genome: *tufA* (apicoplast) 5′-GATATTGATTCAGCTCCAGAAGAAA-3′ / 5′-ATATCCATTTGTGTGGCTCCTATAA-3′, *cytb3* (mitochondria) 5′-AGATACATGCACGCAACAGG-3′ / 5′-TCATTTGACCCCATGGTAAGA-3′, and *CHT1* (nuclear) 5′-TGTTTCCTTCAACCCCTTTT-3′ / 5′-TGTTTCCTTCAACCCCTTTT-3′. Reactions contained template DNA, 0.15 µM of each primer, and 0.75× LightCycler 480 SYBR Green I Master mix (Roche). PCR reactions were performed at 56°C primer annealing and 65°C template extension for 35 cycles on a Lightcycler 6500 (Roche). Relative quantification of target genes was determined using the method of Pfaffl [61]. For each time point, the organelle∶nuclear genome ratio of the antibiotic-treated control or antibiotic-treated, IPP-rescued experiment was calculated relative to that of an untreated control collected at the same time.

### Immunoblot

Ring-stage D10 ACP_L_-GFP parasites from 1 mL of culture were isolated by saponin lysis, washed with PBS, and resuspended in 1×NuPAGE LDS sample buffer (Invitrogen). Proteins were separated by electrophoresis on 12% Bis-Tris gel (Invitrogen) and transferred to nitrocellulose membrane. After blocking, membranes were probed with 1∶1,000 polyclonal rabbit anti-GFP (Clontech) antibody and 1∶15,000 Alexa Fluor 810-conjugated anti-rabbit IgG secondary antibody (Invitrogen). Fluorescent antibody-bound proteins were detected with Odyssey Imager at 800 nm (LiCor Biosciences).

### Fluorescence Microscopy

Untreated and antibiotic treated/IPP rescued D10 ACP_L_-GFP parasites were incubated in 2 µg/mL Hoescht 33342 stain for 30 min at 37°C. Cells in culture media were settled onto 35 mm glass-bottom petri dishes (MakTek) coated with 1% polyethylenimine (Sigma). Widefield epifluorescence live cell images were obtained on a Nikon Eclipse Ti-E equipped with a Coolsnap HQ2 camera (Photometrics) using a 100×/1.4 oil immersion objective. Confocal live cell images were obtained on an A1 confocal mounted on a Nikon Eclipse Ti-E using a 60×/1.4 oil immersion objective. Images were analyzed by NIS-Elements software (Nikon).

## Supporting Information

Figure S1 Fosmidomycin rescue with methylbutenols. Chemical rescue of fosmidomycin inhibition in media supplemented with 0–2 mM 3-methyl-3-butenol (IPP alcohol analog) or 0–2.25 mM 3- and 2-methyl-3-butenol (IPP and DMAPP alcohol analogs).
(TIFF)

Figure S2 IPP rescue of chloroquine inhibition. Chemical rescue of chloroquine inhibition (0–20 µM) in media supplemented with 200 µM IPP.
(TIFF)

Figure S3 Rescue of antibiotic delayed death in strain D10. Survival of D10 parasites over a time course of treatment with chloramphenicol only, chloramphenicol+IPP, and chloramphenicol+IPP for 3 cycles followed by removal of both. Parasitemia is normalized to that of an untreated control. Data from a single experiment are shown.
(TIFF)

Figure S4 Rescue of antibiotic delayed death and apicoplast genome loss with chloramphenicol treatment in strain W2. (A) Survival of W2 parasites over a time course of treatment with chloramphenicol only, chloramphenicol+IPP, and chloramphenicol+IPP for 3 cycles followed by removal of both. Parasitemia is normalized to that of an untreated control. (B) Apicoplast∶nuclear and (C) mitochondria∶nuclear genome ratio of chloramphenicol only and chloramphenicol+IPP treated parasites over the same time course. Genome ratios are normalized to an untreated control. Data from experiments carried out in triplicate are shown.
(TIFF)

Figure S5 Survival of antibiotic-treated, IPP-rescued parasites in media supplemented with 10% human serum. Untreated and antibiotic-treated, IPP-rescued W2 parasites were initially grown in media containing Albumax. Each culture was washed and then split into two cultures, which were resuspended in RPMI media supplemented with either 0.25% Albumax or 10% human serum at a starting parasitemia of 1%–2%. Parasitemia was determined by 200-cell counts of Giemsa-stained blood smears. Growth of the 10% serum culture is shown relative to that of the Albumax culture for both untreated and rescued strains. Data from a single experiment are shown.
(TIFF)

Figure S6 Fosmidomycin rescue with farnesol and geranylgeraniol. Chemical rescue of fosmidomycin inhibition in media supplemented with 0–500 µM farnesol or geranylgeraniol.
(TIFF)

Table S1 Components of RPMI Medium 1640 (Invitrogen), their plasma concentration, and evidence for their acquisition or biosynthesis by blood-stage *Plasmodium*.
(DOC)

Video S1 Movie showing 360° rotation of confocal images of untreated ACP_L_-GFP parasites shown in Figure 4.
(M4V)

Video S2 Movie showing 360° rotation of confocal images of chloramphenicol+IPP treated ACP_L_-GFP parasites shown in Figure 4.
(M4V)

## Abbreviations

DMAPP: dimethylallyl pyrophosphate
GFP: green fluorescent protein
IPP: isopentenyl pyrophosphate
RBC: red blood cell
