# Supplementary figures and images for: Chemical Rescue of Malaria Parasites Lacking an Apicoplast Defines Organelle Function in Blood-Stage Plasmodium falciparum
Source: PLoS Biol. 2011 Aug 30;9(8):e1001138. doi: 10.1371/journal.pbio.1001138 (PMC3166167; doi:10.1371/journal.pbio.1001138)

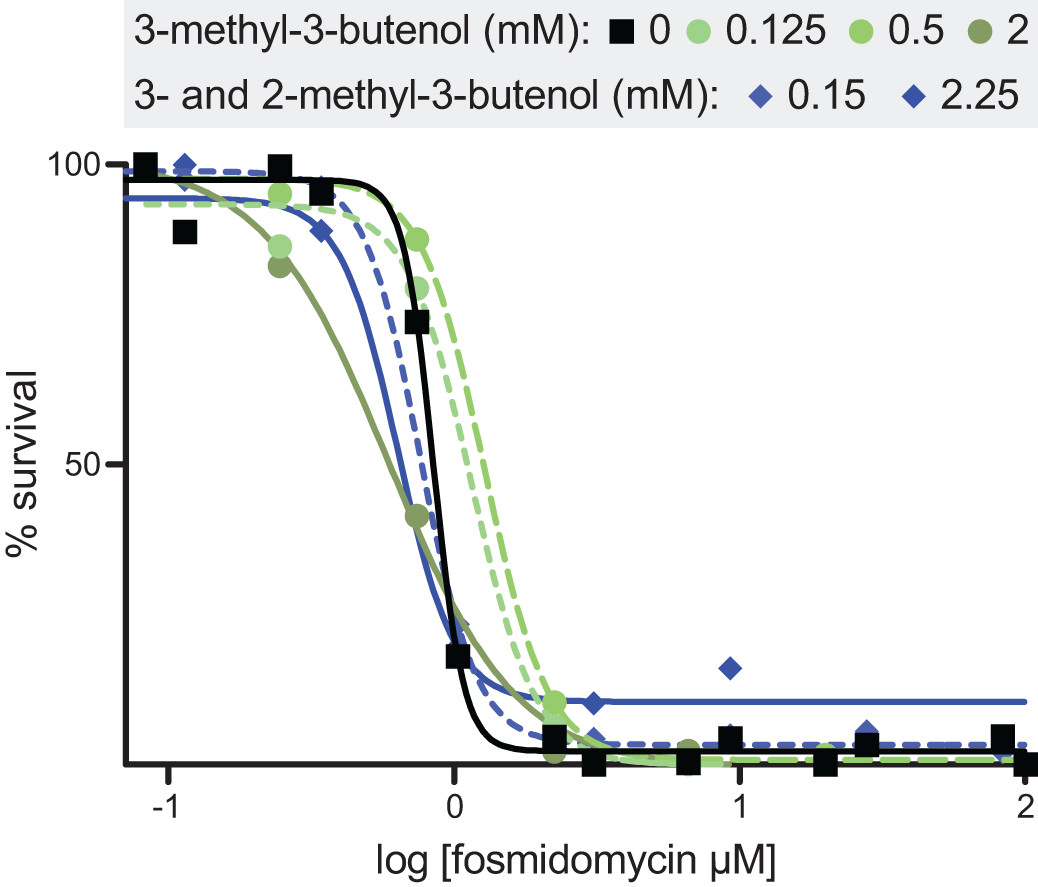

Supplement: Figure S1 — Fosmidomycin rescue with methylbutenols. Chemical rescue of fosmidomycin inhibition in media supplemented with 0–2 mM 3-methyl-3-butenol (IPP alcohol analog) or 0–2.25 mM 3- and 2-methyl-3-butenol (IPP and DMAPP alcohol analogs). (TIFF) [file pbio.1001138.s001.tif]

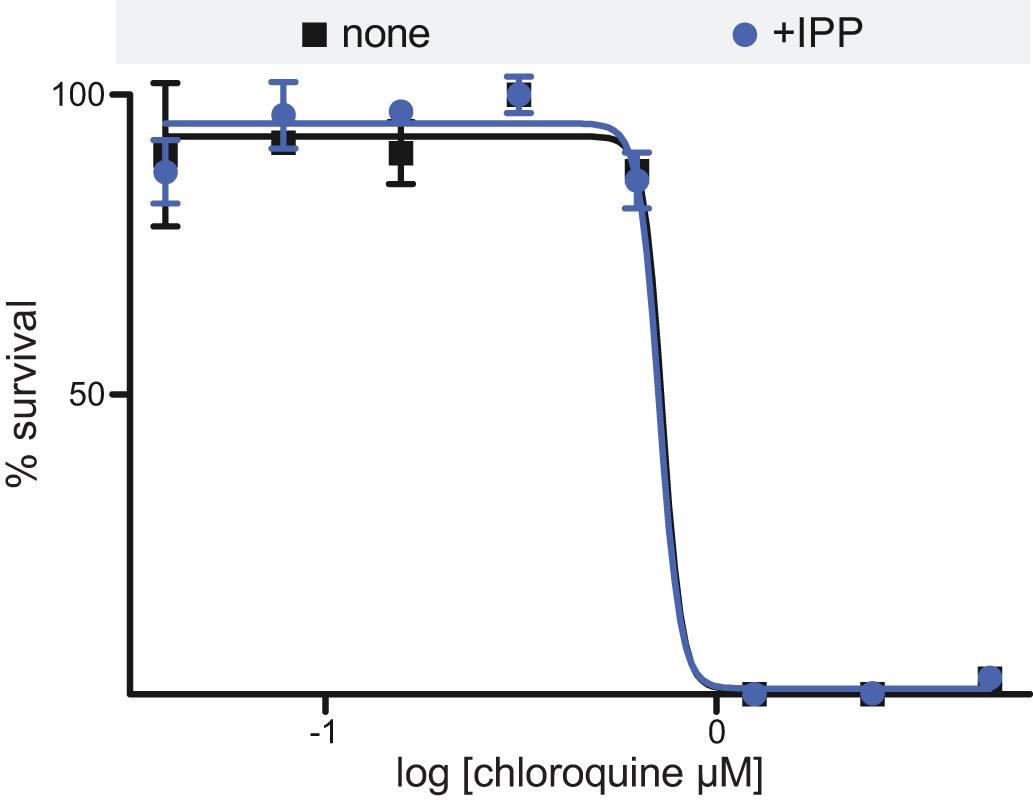

Supplement: Figure S2 — IPP rescue of chloroquine inhibition. Chemical rescue of chloroquine inhibition (0–20 µM) in media supplemented with 200 µM IPP. (TIFF) [file pbio.1001138.s002.tif]

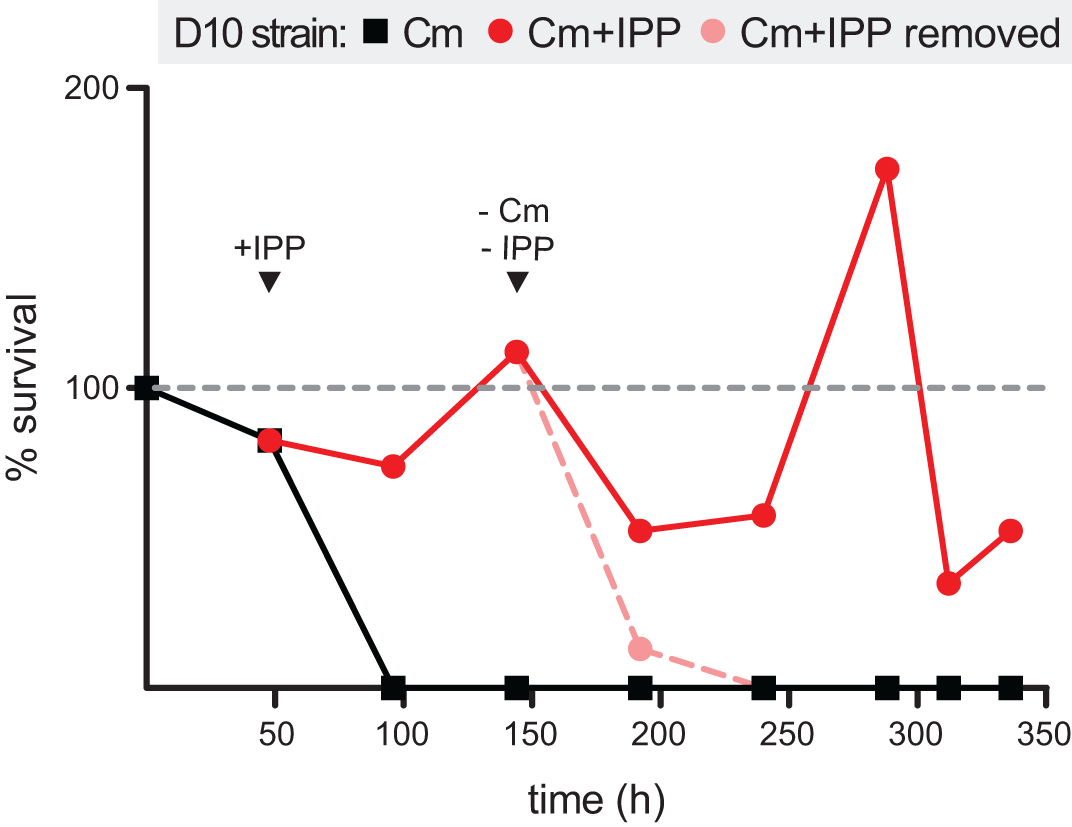

Supplement: Figure S3 — Rescue of antibiotic delayed death in strain D10. Survival of D10 parasites over a time course of treatment with chloramphenicol only, chloramphenicol+IPP, and chloramphenicol+IPP for 3 cycles followed by removal of both. Parasitemia is normalized to that of an untreated control. Data from a single experiment are shown. (TIFF) [file pbio.1001138.s003.tif]

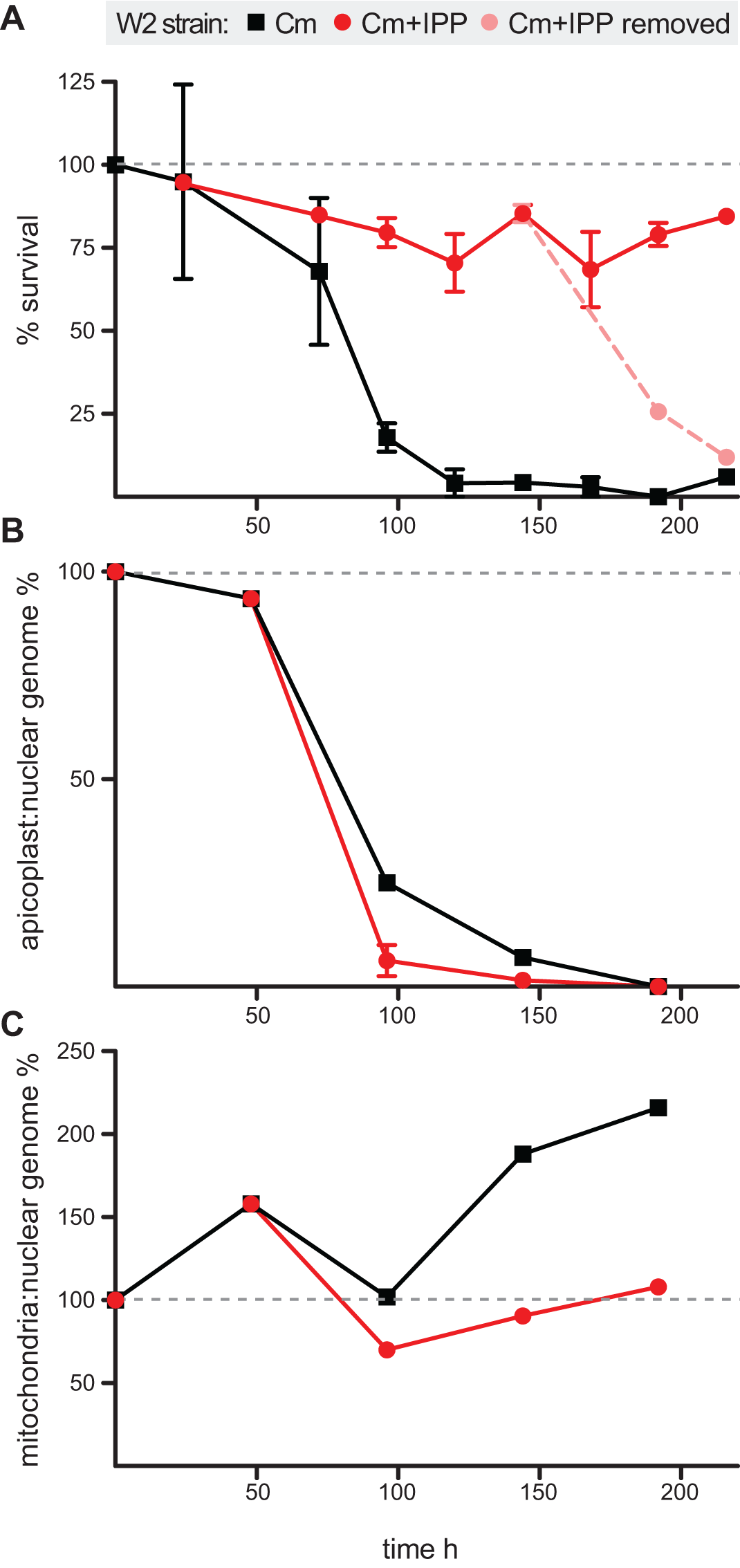

Supplement: Figure S4 — Rescue of antibiotic delayed death and apicoplast genome loss with chloramphenicol treatment in strain W2. (A) Survival of W2 parasites over a time course of treatment with chloramphenicol only, chloramphenicol+IPP, and chloramphenicol+IPP for 3 cycles followed by removal of both. Parasitemia is normalized to that of an untreated control. (B) Apicoplast∶nuclear and (C) mitochondria∶nuclear genome ratio of chloramphenicol only and chloramphenicol+IPP treated parasites over the same time course. Genome ratios are normalized to an untreated control. Data from experiments carried out in triplicate are shown. (TIFF) [file pbio.1001138.s004.tif]

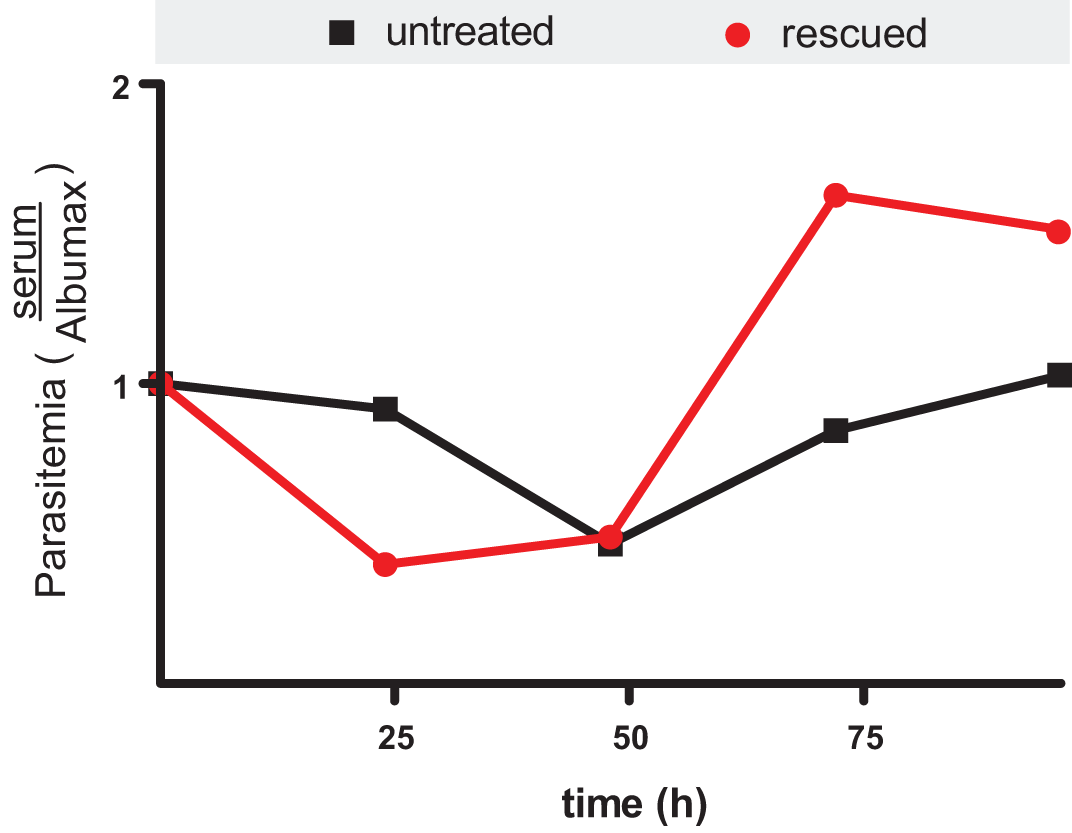

Supplement: Figure S5 — Survival of antibiotic-treated, IPP-rescued parasites in media supplemented with 10% human serum. Untreated and antibiotic-treated, IPP-rescued W2 parasites were initially grown in media containing Albumax. Each culture was washed and then split into two cultures, which were resuspended in RPMI media supplemented with either 0.25% Albumax or 10% human serum at a starting parasitemia of 1%–2%. Parasitemia was determined by 200-cell counts of Giemsa-stained blood smears. Growth of the 10% serum culture is shown relative to that of the Albumax culture for both untreated and rescued strains. Data from a single experiment are shown. (TIFF) [file pbio.1001138.s005.tif]

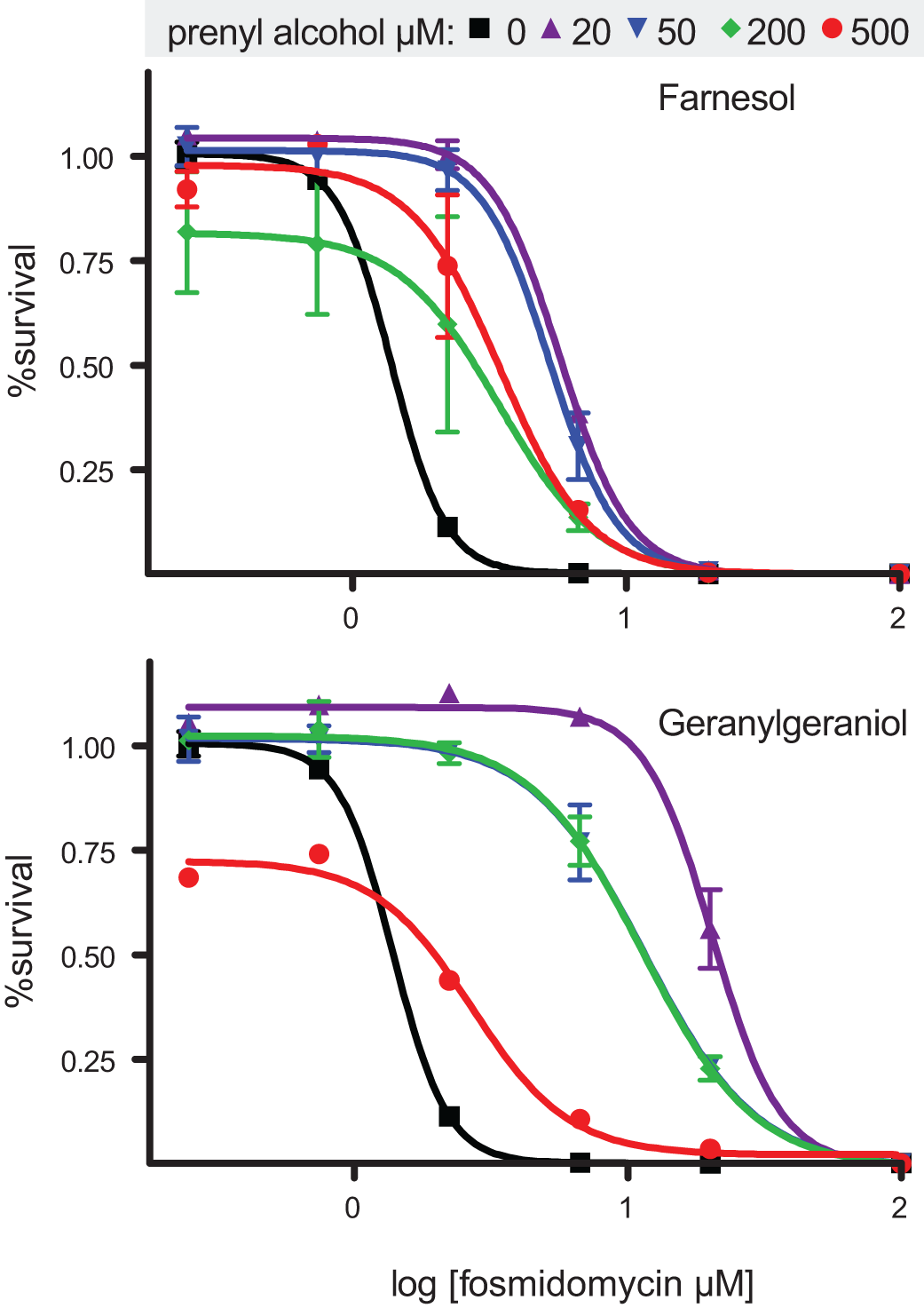

Supplement: Figure S6 — Fosmidomycin rescue with farnesol and geranylgeraniol. Chemical rescue of fosmidomycin inhibition in media supplemented with 0–500 µM farnesol or geranylgeraniol. (TIFF) [file pbio.1001138.s006.tif]
