# Supplementary material for: Chemical Rescue of Malaria Parasites Lacking an Apicoplast Defines Organelle Function in Blood-Stage Plasmodium falciparum
Source: PLoS Biol. 2011 Aug 30;9(8):e1001138. doi: 10.1371/journal.pbio.1001138 (PMC3166167; doi:10.1371/journal.pbio.1001138)
Supplement: Table S1 — Components of RPMI Medium 1640 (Invitrogen), their plasma concentration, and evidence for their acquisition or biosynthesis by blood-stage Plasmodium. (DOC) [file pbio.1001138.s007.doc]

**Table S1. Components of RPMI Medium 1640 (Invitrogen)**

|  | **RPMI** | **Plasma**^1^ | **Acquisition** | **Biosynthesis** |
| --- | --- | --- | --- | --- |
|  | mM | mM |  |  |
| ***Amino Acids**** | | | | |
| **Glycine** | 0.133 | 0.2±0.06 | Hb, plasma^2-4^ | cytosol^5^ |
| **L-Arginine** | 1.15 | 0.1±0.02 | Hb, plasma^2-4^ |  |
| **L-Asparagine** | 0.379 | 0.13±0.008 | Hb, plasma^2-4^ | cytosol^5^ |
| **L-Aspartic acid** | 0.15 | 0.02±0.005 | Hb, plasma^2-4^ | cytosol^2,5^ |
| **L-Cystine** | 0.208 | 0.107±0.02 | Hb, plasma^2-4^ |  |
| **L-Glutamic Acid** | 0.136 | 0.06±0.02 | Hb, plasma^2-4^ | cytosol^2,5^ |
| **L-Glutamine** | 2.05 | 0.5±0.17 | Hb, plasma^2-4^ | cytosol^5^ |
| **L-Histidine** | 0.0968 | 0.09±0.03 | Hb, plasma^2-4^ |  |
| **L-Hydroxyproline** | 0.153 | 0.046±0.046 | plasma^3^ |  |
| **L-Isoleucine** | 0.382 | 0.059±0.03 | plasma^4^ |  |
| **L-Leucine** | 0.382 | 0.25±0.005 | Hb, plasma^2-4^ |  |
| **L-Lysine** | 0.274 | 0.43±0.02 | Hb, plasma^2-4^ |  |
| **L-Methionine** | 0.101 | 0.02±0.01 | Hb, plasma^2-4^ |  |
| **L-Phenylalanine** | 0.0909 | 0.17±0.003 | Hb, plasma^2-4^ |  |
| **L-Proline** | 0.174 | 0.263±0.15 | Hb, plasma^2-4^ | cytosol^5^ |
| **L-Serine** | 0.286 | 0.236±0.006 | Hb, plasma^2-4^ |  |
| **L-Threonine** | 0.168 | 0.26±0.01 | Hb, plasma^2-4^ |  |
| **L-Tryptophan** | 0.0245 | 0.044±0.007 | Hb, plasma^2-4^ |  |
| **L-Tyrosine** | 0.111 | 0.142±0.004 | Hb, plasma^2-4^ |  |
| **L-Valine** | 0.171 | 0.233±0.043 | Hb, plasma^2-4^ |  |
| ***Vitamins*** | | | | |
| **Biotin** | 0.00082 | 0.00127±0.0007 | plasma^6^ |  |
| **Choline chloride** | 0.0214 | 0.011±0.002 | plasma^3^ |  |
| **D-pantothenate*** | 0.00052 | 0.005±0.0004 | plasma^7,6^ |  |
| **Folic acid*** | 0.00227 | 0.00008 | plasma^8,9^ | cytosol^9^ |
| **Nicotinamide*** | 0.0082 | 0.00044 | RBC, plasma^10^ |  |
| **PABA** | 0.0073 | 0.015 | plasma^3^ |  |
| **Pyridoxine*** | 0.00485 | 0.000025 | RBC, plasma^6,10^ | cytosol^6^ |
| **Riboflavin*** | 0.00053 | 0.000015 | RBC, plasma^10^ |  |
| **Thiamine*** | 0.00297 | 0.00012 | RBC^6^ | cytosol^6^ |
| **Vitamin B12** | 3.7E-06 | 0.0000003 | RBC, plasma |  |
| **i-Inositol** | 0.194 | 0.03±0.019 |  | cytosol^11^ |
| ***Other*** | | | | |
| **D-Glucose*** | 11.11 | 4.44±0.37 | plasma^12^ |  |
| **Glutathione*** | 0.00326 | 0.037±0.0048 |  | cytosol^13-15^ |
| **Hypoxanthine*** | 0.10 | 0.005±0.0004 | RBC^16^ |  |
| **HEPES** | 25.03 |  |  |  |
| **Phenol Red** | 0.0133 |  |  |  |

Concentrations of organic metabolites in RPMI medium were obtained from <http://www.invitrogen.com/site/us/en/home/support/Product-Technical-Resources/media_formulation.192.html>. The plasma concentration of each component indicates the availability of the nutrient in the extracellular milieu during human blood infection.^1^ The mode of acquisition from plasma, host RBC, or hemoglobin (Hb) degradation and location of biosynthesis of each nutrient by blood-stage *Plasmodium* is indicated based on available evidence. * denotes nutrients which have been shown to be essential for blood-stage parasites.

**Reference**

1. Psychogios, N. et al. The Human Serum Metabolome. *PLoS ONE* **6**, e16957 (2011).

2. Sherman, I.W. Amino acid metabolism and protein synthesis in malarial parasites. *Bull. World Health Organ* **55**, 265-276 (1977).

3. Kirk, K. Membrane transport in the malaria-infected erythrocyte. *Physiol. Rev* **81**, 495-537 (2001).

4. Liu, J., Istvan, E.S., Gluzman, I.Y., Gross, J. & Goldberg, D.E. Plasmodium falciparum ensures its amino acid supply with multiple acquisition pathways and redundant proteolytic enzyme systems. *Proc. Natl. Acad. Sci. U.S.A* **103**, 8840-8845 (2006).

5. Payne, S.H. & Loomis, W.F. Retention and loss of amino acid biosynthetic pathways based on analysis of whole-genome sequences. *Eukaryotic Cell* **5**, 272-276 (2006).

6. Müller, S. & Kappes, B. Vitamin and cofactor biosynthesis pathways in Plasmodium and other apicomplexan parasites. *Trends Parasitol* **23**, 112-121 (2007).

7. Divo, A.A., Geary, T.G., Davis, N.L. & Jensen, J.B. Nutritional requirements of Plasmodium falciparum in culture. I. Exogenously supplied dialyzable components necessary for continuous growth. *J. Protozool* **32**, 59-64 (1985).

8. Wang, P., Wang, Q., Sims, P.F.G. & Hyde, J.E. Characterisation of exogenous folate transport in Plasmodium falciparum. *Mol Biochem Parasitol* **154**, 40-51 (2007).

9. Hyde, J.E. Exploring the folate pathway in Plasmodium falciparum. *Acta Tropica* **94**, 191-206 (2005).

10. Geary, T.G., Divo, A.A. & Jensen, J.B. Nutritional requirements of Plasmodium falciparum in culture. II. Effects of antimetabolites in a semi-defined medium. *J. Protozool* **32**, 65-69 (1985).

11. Ginsburg, Hagai Malaria Parasite Metabolic Pathways. at <http://sites.huji.ac.il/malaria/>

12. Olszewski, K.L. & Llinás, M. Central carbon metabolism of Plasmodium parasites. *Mol. Biochem. Parasitol* **175**, 95-103 (2011).

13. Ayi, K., Cappadoro, M., Branca, M., Turrini, F. & Arese, P. Plasmodium falciparum glutathione metabolism and growth are independent of glutathione system of host erythrocyte. *FEBS Letters* **424**, 257-261 (1998).

14. Lüersen, K., Walter, R.D. & Müller, S. Plasmodium falciparum-infected red blood cells depend on a functional glutathione de novo synthesis attributable to an enhanced loss of glutathione. *Biochem. J* **346 Pt 2**, 545-552 (2000).

15. Atamna, H. & Ginsburg, H. The malaria parasite supplies glutathione to its host cell--investigation of glutathione transport and metabolism in human erythrocytes infected with Plasmodium falciparum. *Eur. J. Biochem* **250**, 670-679 (1997).

16. Asahi, H., Kanazawa, T., Kajihara, Y., Takahashi, K. & Takahashi, T. Hypoxanthine: A Low Molecular Weight Factor Essential for Growth of Erythrocytic Plasmodium Falciparum in a Serum-Free Medium. *Parasitology* **113**, 19-23 (1996).
